# Supplementary figures and images for: Cholesterol metabolism and intrabacterial potassium homeostasis are intrinsically related in Mycobacterium tuberculosis
Source: PLoS Pathog. 2025 May 22;21(5):e1013207. doi: 10.1371/journal.ppat.1013207 (PMC12136442; doi:10.1371/journal.ppat.1013207)

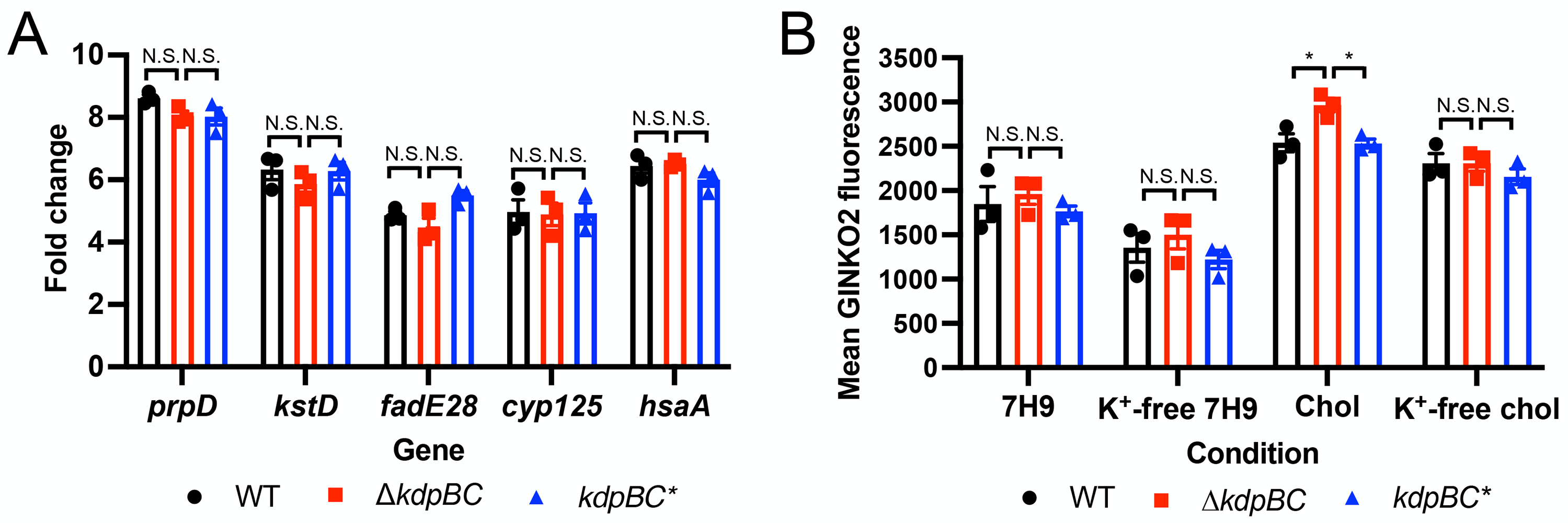

Supplement: S1 Fig — (A) Deletion of kdpBC does not affect Mtb response to cholesterol. WT, ∆kdpBC, and kdpBC* (complemented mutant) Mtb were exposed to 7H9 or cholesterol medium for 4 hours, before RNA extraction for qRT-PCR analysis. Fold change is as compared to the 7H9 condition, with sigA as the control gene. (B) Deletion of kdpBC does not affect intrabacterial [K+] in Mtb. WT, ∆kdpBC, and kdpBC* Mtb each carrying the P606’::GINKO2 reporter were subcultured to OD600 = 0.3 into: (i) 7H9 medium, (ii) K+-free 7H9 medium, (iii) cholesterol medium, or (iv) K+-free cholesterol medium. GINKO2 fluorescence was measured by flow cytometry 6 days post-assay start. Reporter signal from 10,000 Mtb cells per sample per experimental run were obtained for determination of the mean GINKO2 fluorescence for each sample. Data in both panels are shown as means ± SEM from 3 experiments. p-values were obtained with an unpaired t-test with Welch’s correction and Holm-Sidak multiple comparisons in (A), and a two-way ANOVA with Tukey’s multiple comparisons test in (B). N.S. not significant, * p < 0.05. The numerical data underlying the graphs shown in this figure are provided in S1 Data. (TIF) [file ppat.1013207.s001.tif]

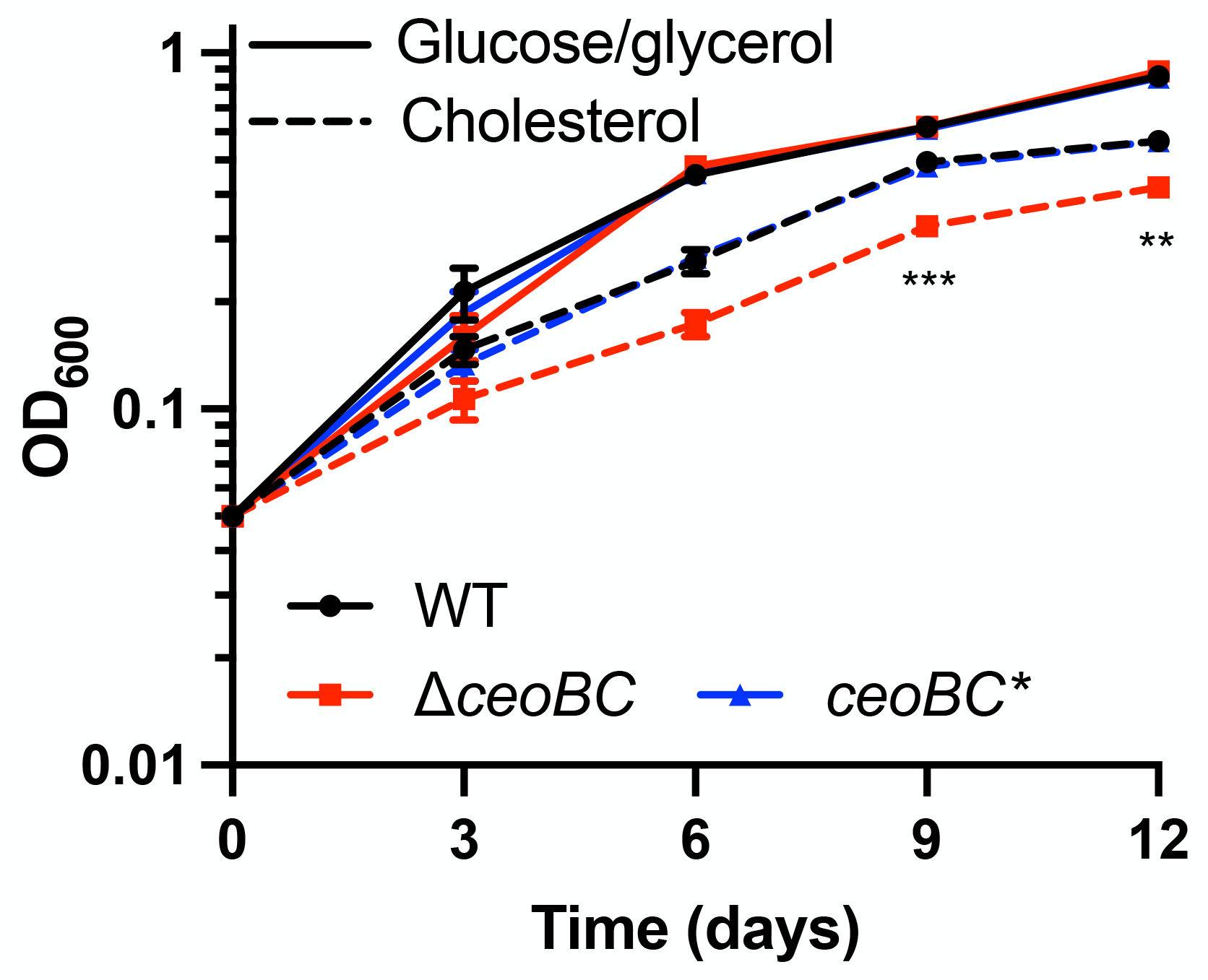

Supplement: S2 Fig — WT, ∆ceoBC, and ceoBC* Mtb were grown in media with either 2 g/l glucose + 0.2% glycerol or 200 µM cholesterol as the sole carbon source, and OD600 monitored over time. Data are shown as means ± SEM from three experiments. Statistical analyses were performed using an unpaired t-test with Welch’s correction and Holm-Sidak multiple comparisons. Comparisons of ∆ceoBC to WT in the cholesterol condition are shown. ** p < 0.01, *** p < 0.001. The numerical data underlying the graphs shown in this figure are provided in S1 Data. (TIF) [file ppat.1013207.s002.tif]

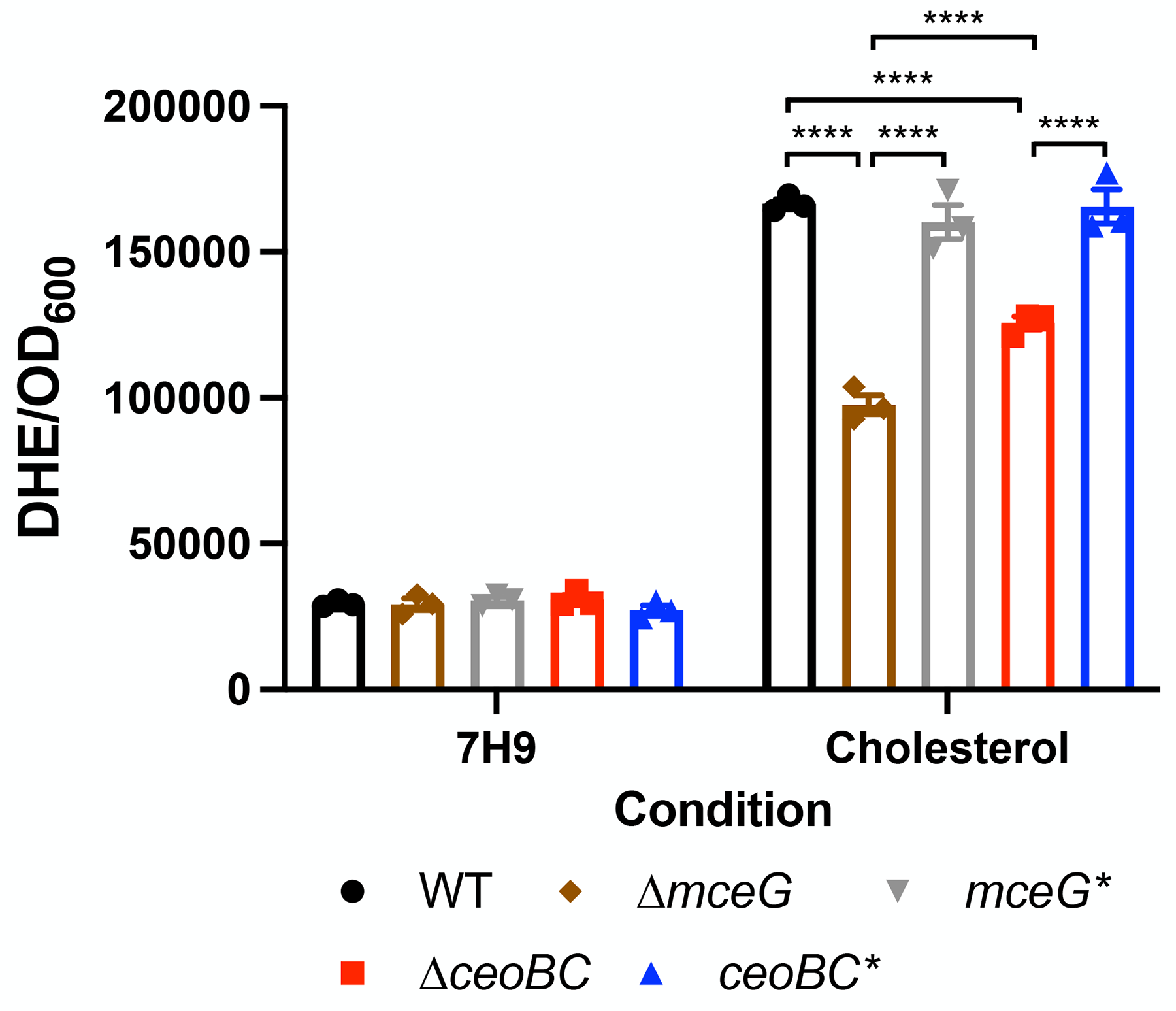

Supplement: S3 Fig — Log-phase WT, ∆mceG, mceG* (complemented mutant), ∆ceoBC, and ceoBC* (complemented mutant) Mtb were exposed to 7H9 or cholesterol media, supplemented with 1 µM dehydroergosterol (DHE), for 24 hours. DHE uptake into Mtb was measured via analysis of DHE fluorescence on a microplate reader, normalized against OD600. Data are shown as means ± SEM from three experiments. p-values were obtained with a two-way ANOVA with Tukey’s multiple comparisons test. All comparisons in the 7H9 condition were non-significant. **** p < 0.0001. The numerical data underlying the graphs shown in this figure are provided in S1 Data. (TIF) [file ppat.1013207.s003.tif]

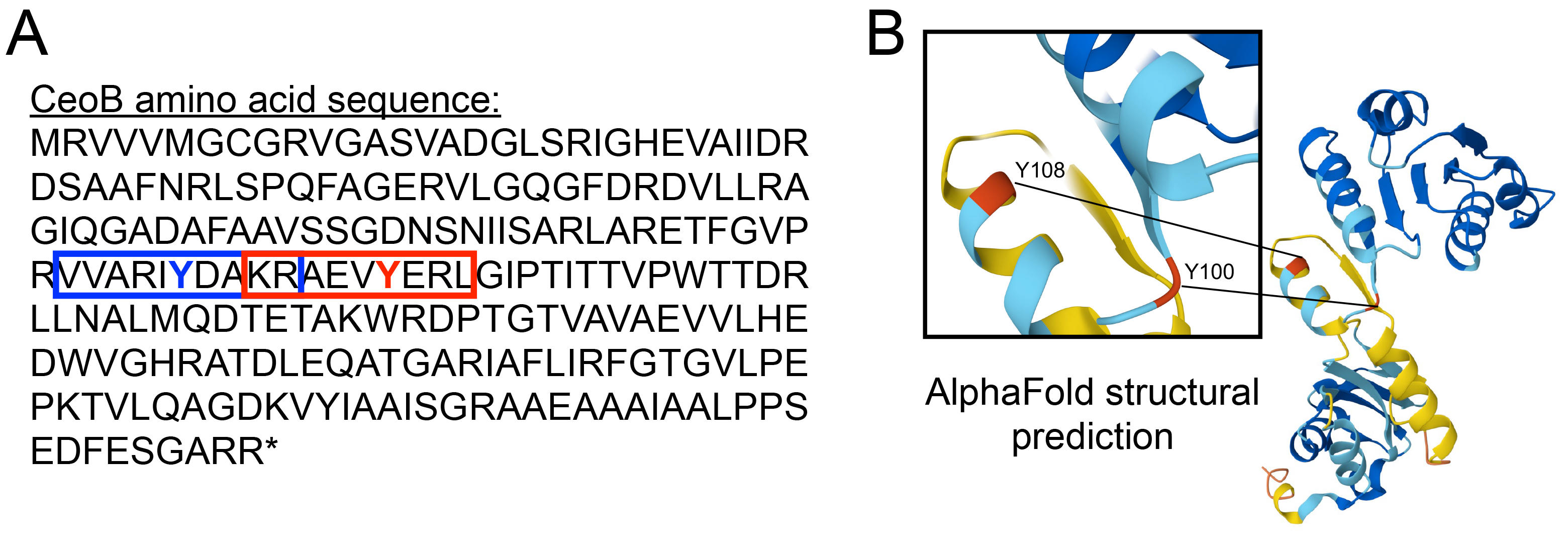

Supplement: S4 Fig — (A) shows the amino acid sequence of CeoB (from the genome of the Erdman strain, accession #AP012340.1 [92]) with the CRAC motif boxed in blue and the CARC motif boxed in red. The key tyrosine residue in each of the motifs are bolded and marked in the AlphaFold-predicted structure [93,94] shown in (B). (TIF) [file ppat.1013207.s004.tif]

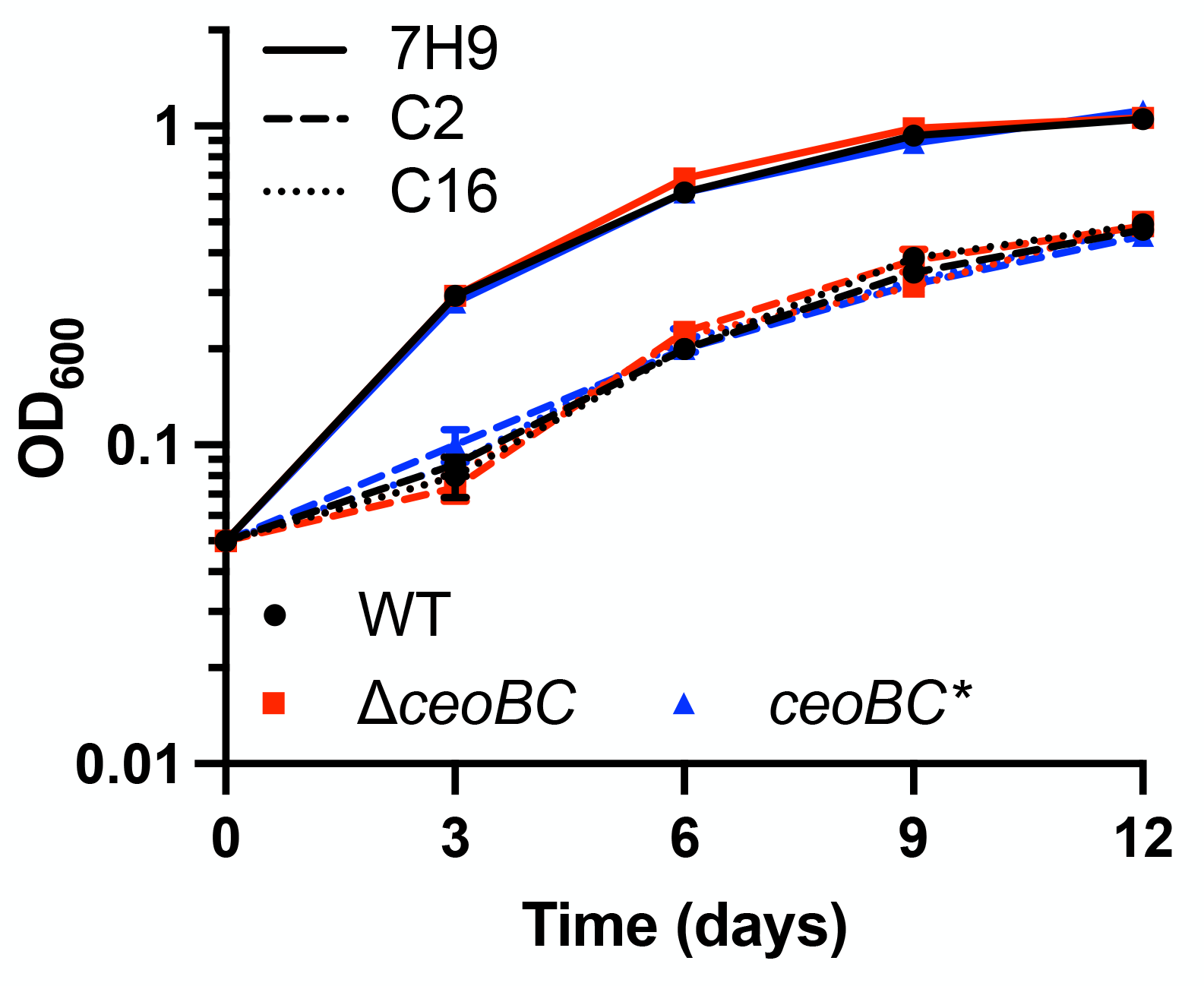

Supplement: S5 Fig — WT, ∆ceoBC, and ceoBC* Mtb were grown in 7H9 or media with acetate (“C2”) or palmitate (“C16”) as a carbon source, and OD600 monitored over time. Data are shown as means ± SEM from three experiments. The numerical data underlying the graphs shown in this figure are provided in S1 Data. (TIF) [file ppat.1013207.s005.tif]
